# Processing Body Image on Social Media: Gender Differences in Adolescent Boys’ and Girls’ Agency and Active Coping

**DOI:** 10.3389/fpsyg.2021.626763

**Published:** 2021-05-21

**Authors:** Ciara Mahon, David Hevey

**Affiliations:** School of Psychology, Trinity College Dublin, Dublin, Ireland

**Keywords:** body image, adolescent(s), social media, body dissatisfaction, positive body image, coping strategies

## Abstract

Although scholars continue to debate the influence of social media on body image, increased social media use, especially engaging in appearance-related behaviors may be a potential risk factor for body dissatisfaction in adolescents. Little research has investigated how adolescents process appearance-related content and the potential strategies they use to protect body image perceptions on social media. To investigate coping strategies used by adolescents, four qualitative focus groups were conducted with 29 adolescents (23 girls) aged 15–16 years (*M* = 15.31, *SD* = 0.47) in mixed-gender Irish secondary schools. Thematic analysis revealed that adolescents employed many different behavioral strategies such as avoiding negative content and selecting positive content. Cognitive processing strategies such as critically evaluating body-related content, psychologically distancing from and positively reframing challenging content were also used, although less frequently. Boys appeared to exhibit greater positive agency over their bodies and social media use and tended to use more active coping styles than girls. Efforts to promote body image on social media such as body positive pages and exposing artificial social media content were considered limited in their effectiveness.

## Introduction

Body dissatisfaction, defined as “a person’s negative thoughts and feelings about his/her body” (Grogan, 1999, p. 2) is a leading cause of eating disorders, disordered eating, low self-esteem and poor psychological wellbeing (Stice and Shaw, 2002; Paxton et al., 2006; Cruz-Sáez et al., 2018). Relatively high prevalence rates of body weight dissatisfaction have been reported cross culturally among adolescent girls [Mean = 48%, Range (26–62%)] and boys [Mean = 31%, Range (15–44%)] in 26 countries (Al Sabbah et al., 2009). Social media is extensively used by adolescents (Pew Research Center, 2018; Rodgers et al., 2020) and has received a lot of research attention as a possible risk factor for body dissatisfaction (Rodgers and Melioli, 2016).

While the causes of body dissatisfaction are considered multifaceted, and include biological, evolutionary, psychological and sociocultural factors (Polivy and Herman, 2002; Ferguson et al., 2011; Fitzsimmons-Craft, 2011), social media is a sociocultural factor that has been suggested by some to be linked to body dissatisfaction. However, the extent to which social media influences body dissatisfaction is debated and the evidence is inconsistent; some studies find associations between social media use and body dissatisfaction (Fardouly et al., 2017; Scully et al., 2020), others find that social media use is associated with positive body image (Cohen et al., 2019), some observe no direct relationships (Ferguson et al., 2014; Cohen et al., 2017) and others suggest that social media may indirectly influence body dissatisfaction by increasing opportunities for other predictors of body dissatisfaction such as peer competition (Ferguson et al., 2014). Furthermore, the inferences that can be drawn regarding social media effects may also be limited by methodological issues in the literature, such as the inability to capture the dynamic, interactive, and personalized nature of social media within a controlled environment or failure to use appropriate controls and procedures to account for demand characteristics (Fardouly and Vartanian, 2016).

Nonetheless, concurring with previous systematic reviews (e.g., Holland and Tiggemann, 2016), a recent meta-analysis of 63 independent samples observed a small, positive, significant relationship between social media use and body image disturbance (Saiphoo and Vahedi, 2019). It should be noted that the meta-analysis’ conclusions are constrained by the literature on which they are based, which as mentioned, has its limitations (i.e., demand characteristics, single-responder bias, common method variance, lack of preregistration, and the fact that many studies report simple bivariate correlations). These limitations may result in an over-estimate of the effect size; consequently, the small effects in this meta-analysis do not necessarily confirm the existence of effects and therefore must be considered as suggestive.^1^ Even though the effect size was small, the authors noted that it is important to further explore the relationship between social media use and body dissatisfaction because social media is extensively used by adolescents. Adolescence is also a particularly vulnerable time for body image (Voelker et al., 2015), and it is important to identify risk/protective factors for body dissatisfaction on social media to help foster more favorable body image during this sensitive developmental period.

Sociocultural theories of body image, such as the Tripartite model (Thompson et al., 1999), propose that social media, influences body image perceptions by conveying messages that emphasize the importance of appearance and pressurize the attainment of unrealistic body ideals. These body-related messages are proposed to give rise to body dissatisfaction directly and indirectly *via* two mediating mechanisms: internalization of and appearance comparisons with body ideals. Body ideal internalization involves endorsing and pursuing body ideals as a personal body standard (Thompson and Stice, 2001), while appearance comparisons involve evaluating one’s appearance relative to others (Jones, 2001). Because the body ideals that individuals internalize are largely unrealistic and unattainable, failure to exemplify these ideals becomes a source of body dissatisfaction when these ideals are valued as a personal goal (Thompson et al., 1999). Upward comparisons, comparisons with “superior” others highlight discrepancies between one’s own body and body ideals thereby giving rise to body dissatisfaction (van den Berg et al., 2002).

Social media are highly visual, appearance focused platforms that extend opportunities to engage in these body dissatisfaction-inducing behaviors (Rodgers and Melioli, 2016). Popular social media platforms used by adolescents such as Instagram and Snapchat (Pew Research Center, 2018), contain a profusion of idealized body related content, which tend to endorse muscular ideals (characterized by a v-shaped torso, visible abs, large biceps, and low body fat,) and lean/athletic ideals (characterized by a toned body with low body fat,) for men/boys. Thin ideals (characterized by a lean physique with low body fat and a narrow waist), fit/athletic ideals (characterized by a lean and muscular physique), and curvy ideals (characterized by a thin waist and large bosom/bottom) are generally more relevant for women/girls (Betz and Ramsey, 2017). Adolescents have been found to endorse and strive for these ideals, despite acknowledging the unrealistic nature of these bodies (Edcoms and Credos, 2016; Burnette et al., 2017; Bell et al., 2019).

Consistent with the Tripartite model, comparisons with celebrities, sports stars, and peers who embody these ideals on social media have been reported by adolescents to give rise to feelings of body dissatisfaction (Edcoms and Credos, 2016; Burnette et al., 2017). Additionally, posting and editing “selfies” (self-portraits of one’s face/body) on social media amplify adolescents’ tendencies to compare and critically evaluate their appearance (Chua and Chang, 2016; Bell, 2019). Adolescent girls tend to engage more in these self-presentation behaviors than boys and tend to be far more invested and influenced by the feedback indices such as “likes” and “comments” received on these posts. Although boys tend not to be greatly affected by the number of “likes” they receive, they are concerned about receiving negative commentary from peers on social media (Kenny et al., 2017).

Some studies suggest that girls’ body image perceptions are more strongly and negatively impacted by social media because they engage with and invest more in body-related content than boys (Frisén and Holmqvist, 2010; McAndrew and Jeong, 2012; Chua and Chang, 2016). Boys have also been found to perceive social media as a more positive, motivating influence on their body image vs. girls who tend to report that social media exerts more negative effects on their body image (Bell et al., 2019). Boys are also thought to be protected somewhat from exposure to aesthetic body ideals, because they value body functionality over aesthetics (Grogan and Richards, 2002). However, recent meta-analyses suggest that the magnitude of social media’s influence on body image is the same for girls and boys (Holland and Tiggemann, 2016; Saiphoo and Vahedi, 2019). It has also been suggested that social media’s impact on male body image may be underestimated because of boys’ tendencies to disclose or downplay body image issues because of stigma surrounding male body image (Griffiths et al., 2014). However, given the methodological issues mentioned previously, the strength of relationship between social media and body image requires more robust examination.

Although appearance-related behaviors on social media have been suggested as a risk factor for body image (Saiphoo and Vahedi, 2019), little research has investigated ways that adolescents manage challenging social media content or strategies they use to buffer the negative effects of these behaviors. It is important to understand the ways that users interact with social media, because the possible body-related outcomes arising from social media use are likely to be the result of complex, reciprocal transactions between the media content and the social media user (Valkenburg and Peter, 2013; Perloff, 2014).

Additionally, while studies have investigated ways to protect and promote adolescent body image in general, social media is a unique sociocultural context that may require specific strategies to help improve body image (Perloff, 2014). Existing approaches to addressing body-dissatisfaction on social media involve teaching social media literacy in order to reduce the credibility of media messages and subsequent body ideal internalization and appearance comparison behaviors (McLean et al., 2017). Although one study found a social media literacy program to be effective in producing gains in body image outcomes in adolescent girls (McLean et al., 2016b), similar improvements were not observed in adolescent boys (Tamplin et al., 2018); this is surprising because it would be anticipated that adolescent boys, who are largely unaware of photo-manipulation/editing of male bodies on social media (Edcoms and Credos, 2016), would benefit from enhanced social media literacy. Although these findings are preliminary, meta-analyses from traditional media literacy interventions indicate that although media literacy programs are effective in increasing knowledge about the media, they do not substantially change body image outcomes (McLean et al., 2016a). This suggests that increasing knowledge about body ideals may not alone be sufficient to address body dissatisfaction and that other strategies/coping tools are required for adolescents to effectively manage problematic appearance-focused social media. Understanding the strategies (if any) that adolescents use can inform the design of interventions such that they target self-protective skills that are in need of cultivation or further development among adolescents. Probing adolescents’ self-protective strategies can also help identify the approaches that might be most effective in improving adolescent body image and can focus intervention efforts toward these.

Only one qualitative study (to the authors’ awareness) with 38 female adolescents aged 12–14 years has explored protective and promotive coping strategies used by adolescents on social media (Burnette et al., 2017). While adolescents in this sample endorsed behaviors associated with body dissatisfaction on social media, including using photo-based platforms, engaging in appearance-related behaviors and making appearance comparisons (Rodgers and Melioli, 2016), they identified several factors that helped protect their body image when using social media. Girls reported that they consciously avoided undesirable social media posts that invoked appearance comparisons or body image concerns as a way of protecting their body image. While this gave adolescents a sense of personal agency over social media use, it was not regarded as a wholly effectual strategy because it was difficult to avoid unsolicited body related content on social media. Participants also evinced high social media literacy levels as they were critical of the body ideals encountered on social media, regarding them as edited, photoshopped, and unrealistic. Participants were also aware of the concerted efforts that peers went to, to capture and post a “perfect” photo of themselves. The authors posited that adolescents’ skepticism and avoidance of idealized body-related content and their appreciation of diverse beauty standards was indicative of protective filtering.

Protective filtering is an aspect of positive body image that involves selectively internalizing messages that promote positive body image and rejecting negative body-related information (Andrew et al., 2015). Protective filtering has been found to buffer the negative effects of exposure to idealized body-related content in the media in adults (Andrew et al., 2015). Protective filtering also appeared to provide promotive benefits to adolescents’ body image in sample of Burnette et al. (2017). However, it is unclear whether the findings of these focus groups are generalizable across adolescents because the sample was relatively small and came from a single-sex, private school that taught social media literacy and critical thinking skills and encouraged an ethos of body appreciation, diversity, and confidence, which was reported to facilitate this protective filtering of social media content. Outside of this study, little research has investigated if adolescents use protective filtering strategies on social media and whether these filtering skills can be fostered in adolescents, including those with negative body image.

It is also not known whether aspects of social media content may help encourage protective filtering; “body acceptance” and “body positive” messages have recently propagated the social media space and have been lauded by adult women as a promising way to buffer against problematic idealized content and decrease body dissatisfaction (Convertino et al., 2019; Rodgers et al., 2019). It is not known whether adolescents engage with this content and whether it exerts protective effects on their body image perceptions (Bell et al., 2017).

Furthermore, little is known about the strategies that adolescent boys use to protect and promote body image. To the authors’ awareness no study has investigated self-protective strategies used by adolescent boys on social media. This reflects a traditional research focus on female body image, as men/boys were thought to be less impacted by body-related issues (Parent, 2013). However, body image has been recognized an increasingly important issue for boys (Parent, 2013), and has been found to be influenced by social media to a similar extent in both boys and girls (Saiphoo and Vahedi, 2019). Boys and girls may face different body-related challenges and pressures on social media (Kenny et al., 2018; Rodgers et al., 2020), and subsequently may employ different strategies to manage these pressures.

This qualitative study explored adolescents’ processing and protective filtering of social media content and whether these strategies were perceived to provide protective benefits for body image. Both adolescent boys and girls were included in the study because little is known about coping or management strategies used, especially by boys, to address gender-specific issues on social media. This study aimed to inform intervention and prevention efforts in the area of body image on social media.

## Materials and Methods

### Design

Focus groups investigated how adolescents managed challenging body-related content and promoted positive body image on social media. Focus groups were used because they provide a rich and ecologically valid insight into the opinions and lived experiences of participants in their own words and from their own perspectives (Greene and Harris, 2011). Focus groups were favored over one-to-one interviews for this kind of exploratory work because they facilitate greater elaboration of ideas and provide a vocabulary to discuss topics (Heary and Hennessy, 2006; Greene and Harris, 2011). In accordance with guidelines (Heary and Hennessy, 2002), single sex focus groups consisting of 6–9 participants were conducted as adolescents have been found to be more comfortable about opening up and discussing sensitive issues in single rather than mixed sex groups.

Focus groups were guided using an interview schedule, which asked adolescents about their experiences and perceptions of body image on social media, the appearance-related challenges they faced on social media and the ways they manage these challenges. The results presented below will focus on adolescents’ management of challenging appearance-related content on social media, however; a brief outline of adolescents’ perceptions/experiences of social media will be provided to contextualize adolescents’ coping strategies. Given the exploratory nature of the research, conversations were allowed to flow freely, and the researcher was free to pursue related topics if they were mentioned.

### Participants

A convenience sample of 29 participants, 23 girls, and 6 boys, aged between 15 and 16 years (*M* = 15.31, *SD* = 0.47) were recruited from two mixed sex Irish secondary schools, one urban private school and one rural community school for a study investigating adolescents’ experiences and perceptions of body image on social media. The study was only open to fourth year students who used social media and who received parental consent to participate. Participants’ ethnicity and other sociodemographic information were not recorded. Four focus groups were conducted, three with girls only and one with boys only (see Table 1).

**Table 1:** Composition and duration of adolescent focus groups.

Group	Gender	School	Size	Duration (min)	Age M (SD) [Range]
1	Female	1	8	39.57	15.38 (0.52) [15–16]
2	Male	1	6	32.23	15.33 (0.52) [15–16]
3	Female	1	6	32.35	15.16 (0.41) [15–16]
4	Female	2	9	49.49	15.33 (0.50) [15–16]

### Materials

Focus groups were audio recorded using an Olympus WS853 voice recorder and qualitative analysis software, MAXQDA (Version 2018.1) was used to analyze the data. The interview schedule included questions such as; (1) What social media activities/behaviors do you think help/harm body image perceptions? (2) What characteristics of social media platforms promote positive body image/negatively impact body image? and (3) How do you manage challenging appearance-focused content on social media?

### Procedure

Full ethical approval was received from the ethics committee at Trinity College Dublin. Permission from school principals was obtained to allow the study to be hosted in schools and for students to participate in the study. Informed consent from parents and informed assent from participants was obtained prior to study commencement. Focus groups were conducted on the school premises and participants were assigned to focus groups based on their class group. Participants’ gender, age, and school attended were obtained in demographic questionnaires that participants completed prior to the focus groups. Focus groups were conducted by two female researchers; the primary researcher led the discussion, while the secondary researcher took notes and kept track of time. Focus groups lasted approximately 30–50 min and participants were offered refreshments, thanked and debriefed afterward.

### Data Analysis

Focus group discussions were transcribed verbatim by the primary researcher (CM) and were analyzed using thematic analysis. The analysis was guided by six step procedure of Braun and Clarke (2006), which involved firstly becoming familiar with the data by transcribing data, reading transcripts and listening to audio recordings (Step 1). Then, initial semantic codes were generated and assigned to the data using MAXQDA software (Step 2). Semantic coding, which involves characterization of explicit, surface meaning of content was deemed the most appropriate form of coding of the personal experiences pertinent to the research question. Data was also coded according to an essentialist/realist perspective, which assumes a unidirectional relationship between meaning and experience. This approach allows for a straightforward exploration of motivations, experiences, and meaning, which were the focus of the research questions. These codes were organized into a coding frame containing concise labels and descriptions for codes was established. Related codes were grouped together to form themes and subthemes (Step 3). An inductive approach, which allows themes to emerge from the data rather than being informed by pre-existing literature, was applied to generate themes (Thomas, 2006). These themes were refined by reviewing the data at the level of the coded extracts and entire data sets to ensure that distinct, coherent themes were generated (Step 4). Themes and subthemes were assigned names and definitions (Step 5).

To verify whether these themes characterized the data, inter-rater agreement was conducted both on codes within the coding frame and final themes identified in the data. As recommended by Breen (2006), an independent researcher (not involved in hosting focus groups) used MAXQDA to review the coded transcriptions and indicate their agreement or disagreement with each of the pre-existing codes and themes; they could also suggest additional codes and themes. The primary researcher reviewed the additional codes/themes suggested by the independent researcher and adjusted coding schemes where appropriate, in consultation with the project lead (DH). According to Breen (2006), to attain adequate consistency (reliability), code-to-sentence matches should occur for at least 80% of cases. Agreement between coders was calculated using the Kappa Coefficient (Brennan and Prediger, 1981) was high, K = 0.92, indicating good inter-rater agreement. Finally, themes were described and contextualized within relevant literature on social media and body image in adolescents (Step 6). These steps were conducted in an iterative, recursive manner.

The researcher adopted a reflexive approach and acknowledged that their own biases and backgrounds shaped the data obtained and the way it was interpreted. The researcher recognized that as a white, Irish, educated woman in her mid-twenties, she could resonate with the struggles of body image and social media pressures to pursue body ideals (insider position) and could recognize that the body-related pursuits and pressures of men/boys and adolescents may differ from her own, and that adolescents’ experience of social media content and affordances may also be divergent (outsider position; Berger, 2015). She also recognized that her adult and female status may have affected adolescents’ interactions and the ways they disclosed information about body image and social media (Berger, 2015; Dodgson, 2019).

## Results

Adolescents reported that they were prolific, habitual users of social media, showing preferences for appearance focused platforms; adolescents, especially girls explicitly reported that they felt social media exerted a mostly negative influence on their body image. Girls strove to attain female body ideals, while boys largely endorsed functionality ideals; appearance comparisons tended to induce body dissatisfaction when these appearance-related goals were not met. Adolescent girls were perceived to invest more in appearance-related behaviors on social media and to experience greater levels body-related pressure, dissatisfaction and self-criticism than boys. Appearance comparisons with peers, social media influencers, and celebrities were identified as the main sources of body dissatisfaction on social media. Thematic analysis revealed two key themes, and various subthemes pertaining to the management of body image on social media by adolescents.

### Theme 1: Behavioral Strategies Used to Manage Problematic Social Media Content

#### Avoidant Strategies

Adolescents reported using avoidant strategies and unfollowing content that contained body-ideals and reducing their social media use. Female 22 “*stopped using [social media as much]*,” while Female 21 “*unfollowed all the celebrities and people with unrealistic body goal standards*” and it was commonly reported that “*not seeing it [social media] as much helped*” (Female 21).

Avoiding social comparisons was emphasized as a core strategy to protect body image. However, some participants felt that avoidance strategies were limited in their effectiveness because it was difficult evade appearance comparisons as body-related images “*were always just popping up*” (Female 2) and body-related content was “*kind of pushed at [them] sometimes*” (Female 2) irrespective of whether they were interested in it or not.

#### Active Selection of Positive Content

Boys believed that they could control the outcomes of social media use by selecting content that promoted their self-image. Boys reported that they “*[did not] really get negative thoughts from looking at [social media], usually [they] just look[ed] at positive stuff*” (Male 4).

However, girls reported that they did not actively select positive content as they felt that all body-related content on social media was damaging. Even content designed to improve body image, such as body-positive content, was viewed skeptically by girls. While girls acknowledged and lauded increased efforts to promote body-acceptance, they held reservations about the effectiveness of these efforts. Participants felt that there was a huge disparity between “*the picture*,” which “*portrays a different message to what it’s captioned*” (Female 22). Participants noted that while a picture may be accompanied by a wholesome caption advocating ostensibly positive messages, the picture itself, which was often appearance/body-focused and objectified, was sending the opposite message.

Female 16 “I think that, what people say when they post something, like what they say might be positive and well-meaning but nearly the pictures themselves speak for themselves and maybe what they are promoting in the pictures isn’t healthy even though they are saying ‘self-love’.”

Participants also found it difficult to endorse messages of body acceptance when they were delivered by individuals who embodied body ideals. Participants found it difficult to reconcile “*See[ing] a very skinny woman and she says ‘love your imperfections’*” (Female 23) because they felt that it was easy for individuals who had perfect bodies to promote the notion of body acceptance as they seemingly had reason to be happy with their bodies. Participants found it difficult to believe that these individuals struggled with body image concerns and thus were reluctant to buy into the notions of acceptance that these individuals were promoting.

Female 7 “A lot of influencers do promote like body confidence and all that but that’s kind of easy for them to say at the same time because they do have the perfect body say for Instagram and all that sort of stuff.”

Other self-acceptance content was recently noted to contain diverse body types including “*plus size models rather than just the really stick thin skinny ones*” (Female 5), which was lauded because it provided a more realistic representation of body image and body types on social media. However, body ideal content with “*skinnier ones [sic: individuals]*” was observed to “*get more positivity back than the plus size ones [individuals] would*” (Female 2) and body ideals were the main attentional draw that influenced bodily self-perceptions. Furthermore, some participants still felt that this body-diversity content reflected extreme body types such as overweight bodies and therefore failed encompass “normal” bodies such as their own.

Female 3 “Nothing’s like normal if you know what I mean.”

Researcher “Right ok, so it’s extremes of all of them kind of?”

Female 3 “Yeah, yeah.”

Researcher “So, nothing in the middle?”

Female 3 “Yeah.”

#### Active Selection of Alternative Platforms

Although girls felt limited in their ability to engage in positive body-related content, especially on Instagram, some girls actively chose to engage with VSCO, an alternative social media platform that was considered less damaging for body image. VSCO was favored because it was not considered to be as “serious” as Instagram and did not contain feedback indices “likes,” “comments” or hierarchical structures such as “followers,” which were problematic features of Instagram. Girls felt that they *“did not feel pressure*” and could post “*a picture on VSCO with no makeup on … but would not put [the same photo] up on Instagram*” (Female 2). Female 1 noted that on “*Instagram ‐ you have to look perfect because you can see how many likes you get and people feel pressured into, they want more likes and that, but you cannot see that on VSCO*.”

VSCO appeared to provide an alternative venue for girls to safely explore their body image without fears of overt judgment from others. However, its use was mentioned by girls in one school, and even among this group Instagram surpassed VSCO in terms of popularity despite the negative effects associated with Instagram.

### Theme 2: Cognitive Strategies

#### Psychological Distancing Strategies

Psychologically distancing oneself from comparison targets was a common strategy utilized by both boys and girls. Focusing on differences between the goals and values of comparison targets vs. themselves served to increase the psychological distance from these targets in boys and lessen their desire or drive to attain these bodies. Male 1 reconciled that “*They’re [celebrities/sports stars/social influencers] kind of devoting their whole life to it*,” while Male 4 concurred “*Yeah that’s their job like*.” Boys felt that they too could attain these ideals if they devoted themselves to this extent but felt secure in their own bodies because they did not hold the same investment or commitment as individuals who possessed body ideals.

Some girls attained psychological distance from targets by focusing on the manipulated, edited nature of the images. Female 10 noted that celebrities/social influencers on social media *“use filters*” and reconciled that “*if [she] used them[filters] [she] would look way better*.*” “Know[ing] that they [celebrities/social influencers] are photoshopped*” helped her to be less affected by them because she knew they were “*unrealistic looking*.” Some girls also attempted to distance themselves from comparison targets by acknowledging that although they often liked the appearance of these individuals, they felt that their features were too extreme and ill-suited to their own appearance.

Female 10 “I like the way they look but I don’t think I’d like to look as … extreme as they do. I don’t think it looks normal. But I think it looks normal with them because they all look like that, but if I walked in like them, I’d look weird, I’d look like an alien.”

While this distancing strategy worked for some, most girls noted that idealized images negatively affected them regardless of the knowledge of their manipulation and this limited the effectiveness of psychological distancing.

#### Reframing Strategies

Both boys and girls reported that reflective practices such as taking a step back, conducting reality checks and looking at the bigger picture enabled them to reassure themselves. Other strategies mentioned by adolescents involved reframing or putting a positive spin on challenging content. One boy suggested that focusing on goals and achievement rather than focusing on discrepancies and feeling self-pity enabled him to process social media content in a healthier way.

Male 4 “Depends on what way you view it really. If you look at it like, saying they’re this and they’re that and I’m just here, you’re not – you’re always just going to be feeling shite like. You are not going to move forward at all. If you just take – just watch whoever, take inspiration, try work yourself, if you want to be like them, work yourself towards being like them.”

In addition, accepting one’s uniqueness and viewing difference as a good rather than negative thing was identified by a female participant as a way of framing body image in a positive light.

Female 21 “I think the problem overall is that we are looking at difference as if it were a problem, we are saying “Why don’t I look like that? Why can’t I be that person?” But I think we all just have to learn to accept that we are all different and we know these facts, but we chose to ignore them!”

Ceasing to judge others and oneself was also mentioned by a few participants, however, it was acknowledged that this was difficult to achieve. Although boys appeared to be less judgmental and more accepting of their bodies with Male 4 noting “*I am grand just the way I am*,” girls struggled to accept their bodies and avoid negative critical self-evaluations, with Female 5 stating “*You have to get a certain amount of likes … or else it’s not like good enough*.”

Female 19 “The more you look at the photo you’re like ‘God I hate it’ you see things that other people wouldn’t see and you’re like ‘I hate everything about it’.”

One girl stressed the value of maintaining a compassionate mindset and endorsed the notion that everyone struggles with the same issues and not to be so harsh and critical toward oneself.

Female 21 “I think we always compare ourselves to the people we see on social media, so we don’t see their flaws, because we are busy pointing out our own in comparison to theirs. We don’t realise that not everyone is perfect as well. And because of that we are kind of blind.”

Female 21 “I just think that young girls need to stop comparing themselves and to take a minute to realise that we are all the same, we are all doing the exact same thing; We are all sitting at home, scrolling. And all the likes we receive, it’s just a double tap of the finger, that person probably doesn’t probably even look at it for more two seconds, we need to stop overthinking everything.”

However, these reframing strategies were only mentioned by a few individuals in focus groups and did not typically reflect the whole groups’ responses to body-related content on social media.

## Discussion

Some participants, particularly girls, reported that they felt social media negatively influenced their body image perceptions. Aligning with the literature, adolescents reported that appearance-focused activities like photo sharing/editing practices and appearance comparisons with celebrities, social media influencers, and peers led to feelings of body dissatisfaction (Edcoms and Credos, 2016; Rodgers and Melioli, 2016; Burnette et al., 2017).

Limiting their social media use and avoiding, unfollowing, or ignoring problematic body related content were the strategies most used by adolescents to protect their body image on social media. However, as found by Burnette et al. (2017), these strategies were considered limited in their effectiveness because of the difficulty in avoiding ubiquitous body-related content on social media. Adolescents were aware of targeted advertising and the fact that their newsfeeds were often propagated with content that they did not necessarily want or chose to see; this limited their perceived control over social media use, especially among girls.

Aligning with these control beliefs, girls tended to report more passive responses to social media such as “putting up” with problematic content. Some boys, on the other hand, reported that they actively sought out and selected positive content that inspired them to exercise or helped them improve in some way. It should be noted that the number of boys in the present study was relatively small. Adolescent girls did not appear to engage in such active selection strategies as they felt that any content related to body image exerted negative effects on them, including content designed to promote positive body image. Adolescent girls’ reservations about body positive/acceptance content is notable as it contrasts with the endorsement of the protective effects of this content for body image in the literature (e.g., Convertino et al., 2019; Rodgers et al., 2019); given the recency of its emergence, the limitations of body positive content may not have been extensively documented in the literature or it may be the case that this kind of content resonates with adult women but not adolescents. Although the influence of body positive content on adolescent body image perceptions requires further research, these findings indicate that adolescent girls experienced social media as a largely negative and disempowering space for body image.

However, VSCO was a photo-sharing platform that was preferred by some girls to Instagram because it did not contain feedback indices such as likes, comments, followers and subsequently did not put as much appearance-related pressure on girls. VSCO has not previously featured in body image research and is worthy of further research attention because it represents a platform that may contain protective features for body image, namely the lack of hierarchical popularity structures or feedback indices.

Some girls distanced themselves from body ideals by reminding themselves that body ideals were not attainable – a strategy also noted by Burnette et al. (2017). However, most girls reported that their knowledge of unrealistic body ideals did little to protect their body image perceptions and they continued to compare despite this awareness. Girls also achieved psychological distance from body ideals by reasoning that while they admired certain body features on others, they did not desire them themselves because these features would be incompatible with their own appearance. Adolescent boys in this sample reported deprioritizing the importance of the muscular ideal and distancing themselves from comparison targets as a way of protecting body image perceptions. This low investment in body-related content was also identified by Holmqvist and Frisén (2012) as a feature that supported adolescent boys’ body image.

Adolescents exhibited a repertoire of strategies to protect and promote body image. The use of these strategies by adolescents and their perceived effectiveness varied. Passive and avoidance strategies were most commonly used but were limited in terms of perceived effectiveness, while active and acceptance strategies were considered effective but were least commonly employed, especially by girls. As these active and acceptance-focused strategies are considered components of positive body image (Holmqvist and Frisén, 2012), enabling adolescents to employ more active cognitive processing and reframing strategies may enhance their resilience to social media content.

Adolescents in this sample did exhibit aspects of protective filtering (as observed by Burnette et al., 2017), in that they were critical of the extreme natures of body ideals and attempted to psychologically distance from and reduce comparisons with these ideals. They also expressed an appreciation of body diversity on social media. However, protective filtering involves both the rejection of negative body-related messages and the endorsement of positive messages (Andrew et al., 2015). Contrasting with the findings of Burnette et al. (2017), high social media literacy levels did not always serve protective effects for body image and adolescent girls in this sample were largely unable to internalize positive body-related messages and struggled to accept/appreciate their own bodies.

Boys appeared to hold more positive perceptions of social media’s influence on body image, processed body-related content in “protective ways” and exhibited higher levels of body-acceptance than girls. Mirroring the findings of the national study of adolescent boys in the United Kingdom of Edcoms and Credos (2016), boys in this sample were less aware of photo-editing and manipulation of images of male bodies on social media and viewed body ideals as attainable with sufficient hard work and effort. It may be the case that social media is experienced as a less pressurizing and more motivating space for boys, encouraging them to hold these more positive evaluations of social media. Alternatively, boys may have deemed it acceptable to report beliefs that body ideals were attainable and that they were not negatively affected by social media in order to adhere to masculine gender roles of self-reliance and dominance (Gattario and Frisén, 2019). Boys may also be less aware of manipulation/editing strategies or less critical in perceptions of body ideal attainability and this might protect them from feelings of disempowerment and dissatisfaction when exposed to body-related content.

Nonetheless, some boys and girls reported self-criticism, self-blame, and body-dissatisfaction from social media comparisons and for perceived failures to adhere to desired body standards. Knowledge/information about body ideals did not always appear to change how individuals felt about their body image. This suggests that enhancing social media literacy and knowledge is not alone sufficient to mitigate tendencies to engage in appearance comparison and body ideal internalization behaviors and help individuals to internalize positive body-related messages. Furthermore, relying on body positive/body acceptance content to promote positive body image is also not sufficient given adolescent girls’ skepticism of this content and its ability to improve their body image perceptions.

Self-compassion approaches are purported to target and change how individuals feel about their bodies by addressing self-criticism and shame at the root of body dissatisfaction (Gilbert and Irons, 2005; Gilbert, 2010). Instead of trying to inhibit appearance comparisons like media literacy approaches, compassion focused approaches (e.g., Neff, 2003; Gilbert, 2009, 2014) try to reduce the self-criticism arising from comparisons – an approach, which may be particularly beneficial in light if the highly self-critical attitudes held particularly by adolescent girls about their bodies. Compassion focused approaches have been found to be effective in reducing body dissatisfaction and disordered eating, in addition to promoting body appreciation and positive body image in adults (Braun et al., 2016; Rahimi-Ardabili et al., 2018). However, the ability of compassion-focused approaches to improve body image outcomes has not been investigated in adolescents (Rahimi-Ardabili et al., 2018).

Compassion-focused approaches may be particularly useful for improving adolescent body image on social media, as they can provide individuals with the skills to reframe self-critical thoughts and enhance their resilience to negative body-related messages on social media. Self-compassion may also enable adolescents, especially girls, to internalize positive body-related messages and foster greater levels of body appreciation (Andrew et al., 2016). They therefore represent a new and potentially promising alternative for tackling body image concerns in adolescents.

### Limitations

Although this study sought to capture a diversity of viewpoints by recruiting from heterogenous schools that differed in terms of school status (private vs. public) and school size (medium-large and small), the sample size of this study was small which limits the transferability of the findings. Furthermore, very few boys participated in the study, which further limits the conclusions that can be made about social media’s influence on their body image perceptions. Due to study, time pressures a pragmatic decision was made to proceed with the analysis and write-up with the imbalanced gender split. This difficulty in recruiting male participants has been noted in the research in this area, and it may be indicative of male stigma around body image and a reluctance among adolescents to discuss it as a topic (Griffiths et al., 2014; Edcoms and Credos, 2016). Future research needs to identify ways of circumventing this stigma and encouraging boys to discuss body image and social media, because far less is known about adolescent boys’ experiences of social media and body image vs. girls, despite the finding that body dissatisfaction is a prevalent and problematic issue among boys and one that is influenced by social media use (Saiphoo and Vahedi, 2019).

The focus group design may have influenced participant’s responses such that they may have provided socially desirable answers that may not have reflected personal opinions, or their opinions may have been swayed by or suppressed because of dominant members of the group.^2^ This may be particularly true of boys, who are less likely to disclose body image concerns because of social norms, which dictate that body image is not an issue for males (Hargreaves and Tiggemann, 2006; Yager et al., 2013). Furthermore, as the focus groups were conducted by female researchers only, boys may have been reluctant to discuss gender differences related to body image (Allen, 2005), while girls, may have been more expressive of their concerns because they tend to prefer same-sex female facilitators (Yager et al., 2013).

### Conclusion

Some adolescents, especially girls, indicated that social media led them to feel dissatisfied with their bodies. Boys and girls appeared to employ different strategies to manage to address the gender-specific challenges they encountered online. Boys appeared to exhibit more agency and active coping strategies, which contrasted with girls who were less optimistic about their ability to control social media outcomes and who struggled to interpret body-related information in a positive, self-protective way. Future research should examine these gender differences in larger samples across diverse contexts.

## Data Availability Statement

The raw data supporting the conclusions of this article will be made available by the authors, without undue reservation.

## Ethics Statement

The studies involving human participants were reviewed and approved by School of Psychology, Trinity College Dublin. Written informed consent to participate in this study was provided by the participants’ legal guardian/next of kin.

## Author Contributions

CM conceived, planned, and carried out the study, analyzed the data, and wrote the manuscript with input from DH, who was involved in the planning and supervision of the study. Both the authors contributed to the article and approved the submitted version.

### Conflict of Interest

The authors declare that the research was conducted in the absence of any commercial or financial relationships that could be construed as a potential conflict of interest.

## Footnotes

^1^We wish to thank a reviewer of the paper for highlighting this issue.

^2^We would like to thank the reviewer for highlighting that demand characteristics may have influenced participant responses such that respondents are likely to just go with the theme of the questions, rather than spontaneously come to the conclusion that social media influences them in negative ways.
